# Multi-algorithms analysis for pre-treatment prediction of response to transarterial chemoembolization in hepatocellular carcinoma on multiphase MRI

**DOI:** 10.1186/s13244-023-01380-2

**Published:** 2023-02-28

**Authors:** Mingzhen Chen, Chunli Kong, Enqi Qiao, Yaning Chen, Weiyue Chen, Xiaole Jiang, Shiji Fang, Dengke Zhang, Minjiang Chen, Weiqian Chen, Jiansong Ji

**Affiliations:** 1 Key Laboratory of Imaging Diagnosis and Minimally Invasive Intervention Research, Lishui Hospital of Zhejiang University, School of Medicine, Lishui, 323000 China; 2 Institute of Imaging Diagnosis and Minimally Invasive Intervention Research, The Fifth Affiliated Hospital of Wenzhou Medical University, Lishui, 323000 China; 3 Clinical College of the Affiliated Central Hospital, School of Medicine, Lishui University, Lishui, 323000 China

**Keywords:** Hepatocellular carcinoma, Transarterial chemoembolization, Deep learning, Radiomics

## Abstract

**Objectives:**

This study compared the accuracy of predicting transarterial chemoembolization (TACE) outcomes for hepatocellular carcinoma (HCC) patients in the four different classifiers, and comprehensive models were constructed to improve predictive performance.

**Methods:**

The subjects recruited for this study were HCC patients who had received TACE treatment from April 2016 to June 2021. All participants underwent enhanced MRI scans before and after intervention, and pertinent clinical information was collected. Registry data for the 144 patients were randomly assigned to training and test datasets. The robustness of the trained models was verified by another independent external validation set of 28 HCC patients. The following classifiers were employed in the radiomics experiment: machine learning classifiers k-nearest neighbor (KNN), support vector machine (SVM), the least absolute shrinkage and selection operator (Lasso), and deep learning classifier deep neural network (DNN).

**Results:**

DNN and Lasso models were comparable in the training set, while DNN performed better in the test set and the external validation set. The CD model (Clinical & DNN merged model) achieved an AUC of 0.974 (95% CI: 0.951–0.998) in the training set, superior to other models whose AUCs varied from 0.637 to 0.943 (*p* < 0.05). The CD model generalized well on the test set (AUC = 0.831) and external validation set (AUC = 0.735).

**Conclusions:**

DNN model performs better than other classifiers in predicting TACE response. Integrating with clinically significant factors, the CD model may be valuable in pre-treatment counseling of HCC patients who may benefit the most from TACE intervention.

## Key points

- DNN and LASSO models performed better than other classifiers in TACE response prediction.
- CD model achieved an AUC of 0.974 in the training set, superior to other comprehensive models.
- CD model may serve as a potential tool for the selection of suitable TACE candidates.

## Introduction

Hepatocellular carcinoma (HCC) is the sixth most common malignant tumor and the third leading cause of cancer-related death worldwide [1]. HCC is characterized by insidious onset, high malignancy, and rapid progression. Hence, up to 70% of the patients are in the intermediate to advanced stages when clinically diagnosed, and less than 20% have surgical indications [2, 3]. For these patients with advanced HCC, transarterial chemoembolization (TACE) has become the first-line primary or adjuvant clinical treatment strategy. Several randomized controlled trial (RCT) studies have demonstrated that TACE can delay tumor progression to varying degrees, thereby providing a potential surgical resection opportunity for patients with initially unresectable HCC [4–6]. However, the tumor response to TACE varies from patient to patient due to the highly heterogeneous tumor biological behavior, such as differences in gene expression, vascular invasion status, and tumor size [7]. Effective TACE can benefit patients, while unproductive treatment would increase the burden on patients and cause waste of medical resources. Therefore, it is crucial to preoperatively select appropriate patients for TACE treatment, and a precise model for predicting response to TACE therapy is desirable.

Clinically, magnetic resonance imaging (MRI) as a routinely used technique in cancer diagnosis provides a non-invasive way to analyze HCC [8]. Radiomics is a promising and easy-to-use modality that involves quantitative features from radiology images [9, 10]. Recent studies have shown that features extracted from liver MR images were related to microvascular invasion (MVI) [11] and showed predictive power on TACE response [12–14] for HCC patients. Typically, radiomics research includes a four-step workflow, with the construction and assessment of a single mathematical model considered as the last step in the procedures [15, 16]. However, sometimes the proposed model is not good enough, for the highest area under the curve (AUC) of most trained models did not reach 0.9 [13, 14]. Hence, some researchers turn to more advantageous algorithms, such as machine learning and deep learning, to achieve the optimization and improvement of models in the field of oncology [17–19]. Specifically, artificial intelligence (AI) techniques are assumed to be a potential tool for precise clinical management and decision-making in HCC patients treated with TACE [20–22]. The continuous collection of medical data and improvement in AI technology are offering researchers the ability to construct models that take various predictors of HCC treatment evaluation into account.

However, it remains unknown which algorithm is the most efficient and optimal, and there are scarce studies that have compared multiple classifiers. Thus, the primary aim of this study was to compare four forecasting models in terms of their accuracy in predicting TACE response before intervention for HCC patients. The four forecasting models include machine learning classifiers k-nearest neighbor (KNN), support vector machine (SVM), the least absolute shrinkage and selection operator (Lasso), and deep learning classifier deep neural network (DNN). The secondary aim was to integrate these classifiers separately with clinical prognostic factors and produce the most powerful comprehensive model.

## Materials and methods

### Study sample

The study has been approved by the Institutional Review Board. Due to the retrospective nature of this study, informed consent was not required. We retrospectively identified all consecutive patients who underwent TACE for HCC from April 2016 to June 2021 in one center. Our inclusion criteria included patients with HCC who underwent initial TACE and had contrast-enhanced MR(CE-MR) before and after TACE, and with complete clinical information (i.e. demographics, preoperative hepatitis, serum alpha-fetoprotein (AFP) levels, and liver function tests). Exclusion criteria included underage patients; synchronous therapies during follow-up time, such as resection, and systemic chemotherapy; other concurrent malignancies and follow-up for less than 3 months post-procedure. HCC was diagnosed histologically or by MR image evaluation. In total, 144 treatment-naïve HCC patients (Median follow-up time, 13.8 weeks) met the inclusion criteria. To further validate the generalization capability of the founded models, we collected 28 HCC patients treated with TACE between August 2021 to October 2022 as an independent external validation set. The inclusion and exclusion criteria of these patients were consonant with the preceding dataset.

### TACE procedure and reference standard of TACE response

All patients included were treated with TACE, including conventional TACE (cTACE) and drug-eluting bead TACE (DEB-TACE). Interventional physicians choose cTACE or DEB-TACE based on tumor burden and patient characteristics. The basic treatment process of DEB-TACE resembles that of cTACE except for the embolic agents. cTACE uses lipiodol (Guerbet), gelatin sponge particles, and polyvinyl alcohol as embolic agents. Selective or super-selective embolization of tumor-supplying vessels is performed whenever technically justified [23]. For DEB-TACE, 100–300 μm diameter CalliSpheres® Beads (CB; Jiangsu Hengrui Pharmaceutical Co., Ltd.) were used as carriers, loaded with 60–80 mg epirubicin, pirarubicin, or doxorubicin. All procedures were administered by interventional physicians with at least 10 years of experience. All patients were admitted for postoperative supportive care after TACE procedure and were managed routinely.

Study cohort judgment of TACE response was performed according to the modified Response Evaluation Criteria in Solid Tumors (mRECIST) [24] criterion. In brief, the therapeutic response of TACE was stratified into four grades: (a) complete response (CR): complete disappearance of the lesion; (b) partial response (PR): a minimum 30% reduction in the sum of diameters of viable target lesions (enhancement in the arterial phase); (c) progressive disease (PD): at least 20% extension in the sum of the diameters of viable (enhancing) target lesions; and (d) stable disease (SD): neither PR nor PD. Based on mRECIST, CR and PR patients were categorized as objective response (OR) cohort, and PD and SD patients as non-objective response (NOR) group. This assessment was determined by two professional abdominal radiologists based upon the follow-up MR images. Among the 144 patients enrolled, 75 were assigned to the NOR group and 69 to the OR group. In the independent external validation set, 14 patients were in the NOR group and 14 in the OR group.

### MRI image acquisition

Before and after TACE, all recruited patients underwent Gadolinium injection meglumine-enhanced MR imaging using 1.5-T and 3.0-T MR scanners. For the Philips ENGENIA 3.0-T MR scanner (Philips Medical Systems), imaging sequences included axial T2-weighted sequence with spectral presaturation with inversion recovery, breath-hold precontrast and post-contrast (after injection 0.1 mmol/kg of Gadopentetate dimeglumine (Gd-DTPA)) mDIXON-T1-weighted (water) sequence and breath-hold diffusion-weighted echo-planar sequence. The main image acquisition parameters were as follows: T2-weighted sequence, repetition time (TR) 3000 ms, echo time (TE) 200 ms, matrix: 200 × 195, thickness 7 mm, gap 1 mm; T1-weighted with breath-hold, TR 3.6 ms, TE1/TE2: 2.38/4.76 ms, matrix: 224 × 166, thickness 5 mm, gap  2.5 mm, field of view (FOV): 400 mm × 314 mm, and 4 dynamic phases were scanned, which were the hepatic arterial phase (AP) (25–30 s), portal venous phase (PVP) (60–70 s), delayed phase (DP) (180 s), and hepatobiliary phase (HBP) (20 min); diffusion-weighted echo-planar sequence, TR 2500 ms, TE 64 ms, thickness 7 mm, gap 1 mm, FOV: 400 × 343 mm, matrix: 116 × 97, *b* value 0, and 800 s/mm^2^.

For the German MAGNETOM Area 1.5 T MR scanner, the MRI scan sequences included: T2-weighted sequence: TR 3500 ms, TE 90 ms, FOV 380 mm × 380 mm, matrix 320 × 320; CE-MR scans were performed with three-dimensional volume interpolation (3D-VIBE): TR 4.1 ms, TE 1.8 ms, FOV: 380 mm × 380 mm, matrix: 320 × 320, thickness 5 mm, gap1 mm. After injecting contrast agent Gd-DTPA (dose 0.1 mmol/kg, flow rate 2 ml/s), the images of AP, PVP, and DP were collected at 25 s, 60 s, and 180 s, respectively.

### Image segmentation and radiomic features

**Fig. 1:**
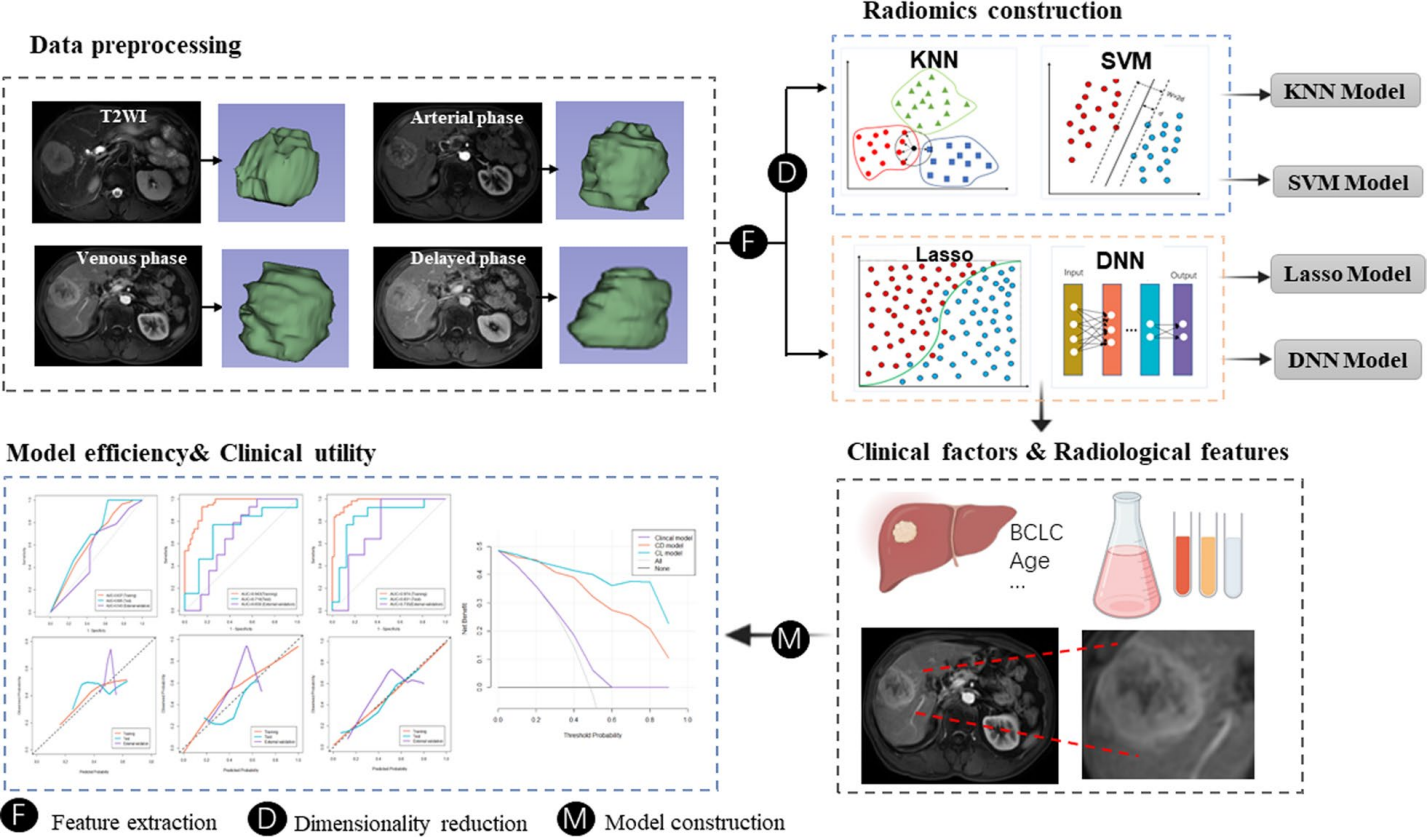
Flowchart of the study procedure. Abbreviation: KNN, k-nearest neighbor; SVM, support vector machine; Lasso, the least absolute shrinkage and selection operator; DNN, deep neural network

The flowchart of the study is depicted in Fig. 1. The volumes of interest (VOIs) of tumors were delineated manually using 3D Slicer version 4.10 (www.slicer.org) by reader 1 (radiologist with 3 years of abdominal imaging experience) and reader 2 (radiologist with 10 years of abdominal neoplasms). The VOIs were drawn on T2-weighted images and 3 dynamic enhanced phase images (namely AP, PVP, and DP). The radiologists involved in the segmentation were unaware of all clinical and prognostic information. To standardize the voxel spacing and control image noise, all images were resampled to a 1 × 1 × 1 mm^3^ voxels with a fixed bin width of 25. Radiomics features were extracted automatically for the T2-weighted images and 3 enhanced phase images by using the PyRadiomics toolkit [25]. For each sequence, 110 radiomic features were extracted automatically. Hence, a total of 440 quantitative features were extracted in this procedure.

To assess the variability of extracted features, 25% of all the involved cases were randomly picked and were again delineated independently by reader 1 (test–retest variability) and reader 2 (interobserver variability). The second lesion segmentations were conducted 2 months after the first segmentations. The intraclass correlation coefficient (ICC) was used to elaborate test–retest and interobserver repeatability, an ICC greater than 0.75 indicated good reproducibility.

### Four forecasting models

This experiment compared the forecasting capability in four models, including machine learning classifiers KNN, SVM, Lasso, and deep learning classifier DNN. The schematic diagram of each algorithm is shown in Figure 1. All previously mentioned radiomics features were standardized using Z-score before model training. To reduce redundant features and prevent reduce bias or over-fitting, the minimum redundancy maximum correlation (mRMR) method was used for dimensionality reduction in KNN and SVM models. Finally, 10 features were retained for constructing the models. Since Lasso and DNN can reduce the dimension of features in an automatic and non-prioritized manner during model training, no additional feature selection methodology was needed.

The first prediction model applied in this study was the KNN algorithm, an instance-based learning method that uses the k-nearest to categorize unknown data of the new sample [26]. In the experiment, the number of neighbors of KNN is 4. The second predictive model used in this study was SVM, which is a supervised algorithm that separates the feature space into hyperplanes based on the object classes [27]. SVM also uses a kernel function to distinguish nonlinearly separable classes. The kernel function of SVM is Radial Basis Function, and the gamma is 0.2. Hence, the SVM algorithm supports both linear and nonlinear classification.

The third forecasting model used in this study was Lasso [28], which can achieve both data dimensionality reduction and feature selection. Based on the linear equations of the respective coefficients of the selected features, Lasso model was established and the Lasso score associated with each patient was obtained. The fourth forecasting model was DNN [29], which is an artificial neural network with multiple layers between the input features and output predictions. Each linear layer in DNN model is connected by nonlinear activation functions to learn complex nonlinear relationships. In this research, we utilized the neural network with BatchNorm and Dropout modules for better performance. BatchNorm [30] is a mini-batch normalization function that can prevent network over-fitting and accelerated training. Dropout [31] is a regularizing tool that randomly drops neurons from the neural network during training. The number of network layers is three and the number of nodes is 440-220-2 per layer. Each layer of the network is connected by a Rectified Linear Unit (ReLU) activation function, and the dropout rate is 0.5. The final activation of the output uses a softmax function to produce scores between 0 and 1. In the DNN experiment, the cosine annealing learning rate is used, and the learning rate is set to 0.01. All the trainable parameters are optimized by Adam algorithm, batch size is 32, and the network is trained for 200 epochs.

### Construction and validation of comprehensive models

For the clinical factors, univariate and multivariate logistic regression analyses were applied to determine the independent predictors of TACE response in the training set. Multimodal features including forementioned classifier outputs (corresponding output values) and clinicopathological variables were incorporated into comprehensive model using the multivariate logistic regression analysis.

The discriminative ability of the predictive model was tested by ROC curve based on the AUC, sensitivity, and specificity. Calibration curves were drawn to compare the probability of TACE response between the predicted and actual rates. Comparisons of the AUCs of the ROC curves were performed using the Delong test. To determine the clinical value of the model, decision curve analysis (DCA) was performed to reckon the net benefits under different threshold probabilities.

### Statistical analyses

Statistical analyses were performed using SPSS v25.0, R v4.0.4. and Python v3.7.6. The Python packages used for KNN, SVM, and Lasso modeling were sklearn.neighbors.KNeighborsClassifier, sklearn.svm.SVC, sklearn.linear_model.Lasso, respectively (sklearn machine learning library version is 1.0 [32]). The deep learning DNN modeling was conducted on the pytorch platform (version 1.10.0). The 144 involved patients were randomly divided into training set and test set with a ratio of 8:2. The differences in patient characteristics data between the OR and NOR groups were assessed for both training and test sets. To identify significant (*p* < 0.05) predictors for TACE response, continuous variables were analyzed using *T* test or Mann–Whitney *U*-test according to the results of Kolmogorov–Smirnov test; categorical variables were analyzed using Chi-square test or Fisher exact analysis. All statistical tests were two-sided; a *p* value ≤ 0.05 was considered statistically significant.

## Results

### Patient characteristics

Table 1 shows the univariate analysis results of demographic, clinical characteristics, and MR imaging features between NOR and OR groups. Of the 144 included patients, 124 patients (86.11%) were men and 20 (13.89%) were women. In the training set, 48.6% of the patients (56 of 115) had OR outcome. Similarly, 44.8% of the patients (13 of 29) had OR outcome in the test set. Indicators such as Child–Pugh classification (*p* = 0.05), and portal venous invasion (*p* = 0.025) illustrated statistical difference between NOR and OR patients; therefore, these characteristics were submitted to subsequent models.

**Table 1:** Clinical characteristics of patients in the training and test sets

	Training set (*n* = 115)		*p* value	Test set (*n* = 29)		*p* value
	NOR	OR		NOR	OR	
Age (years)	59.42 ± 12.54	61.29 ± 11.62	0.411^a^	55.25 ± 11.67	62.538 ± 15.09	0.154^a^
Sex			0.904			0.488
Male	50 (84.7%)	47 (83.9%)		14 (87.5%)	13 (100%)	
Female	9 (15.3%)	9 (16.1%)		2 (12.5%)	0 (0.0%)	
Hypertension			0.392			0.78
No	50 (84.7%)	44 (78.6%)		16 (100%)	10 (76.9%)	
Yes	9 (15.3%)	12 (21.4%)		0 (0.0%)	3 (23.1%)	
Diabetes			0.743			1
No	53 (89.8%)	52 (92.9%)		15 (93.8%)	12 (92.3%)	
Yes	6 (10.2%)	4 (7.1%)		1 (6.2%)	1 (7.7%)	
HBsAg			0.51			1
Negative	18 (30.5%)	14 (25%)		9 (15.3%)	9 (16.1%)	
Positive	41 (69.5%)	42 (75%)		50 (84.7%)	47 (83.9%)	
Child–Pugh classification			0.05			0.697
A	39 (66.1%)	46 (82.1%)		11 (68.8%)	10 (76.9%)	
B/C	20 (33.9%)	10 (17.9%)		5 (31.2%)	3 (23.1%)	
BCLC stage			0.089			0.143
A	32 (54.2%)	39 (69.6%)		4 (25%)	7 (53.8%)	
B/C	27 (45.8%)	17 (30.4%)		12 (75%)	6 (46.2%)	
Treatment modality			0.118			0.379
c-TACE	37 (62.7%)	27 (48.2%)		6 (37.5%)	7 (53.8%)	
DEB‑TACE	22 (37.2%)	29 (51.8%)		10 (62.5%)	6 (46.2%)	
*Laboratory values*						
AFP			0.698			0.606
≤ 400 (ng/ml)	42 (71.2%)	38 (67.9%)		13 (81.2%)	12 (92.3%)	
> 400 (ng/ml)	17 (28.8%)	18 (32.1%)		3 (18.8%)	1 (7.7%)	
CEA			0.251			0.606
≤ 5 ng/ml	52 (88.1%)	45 (80.4%)		13 (81.2%)	12 (92.3%)	
> 5 ng/ml	7 (11.9%)	11 (19.6%)		3 (18.8%)	1 (7.7%)	
AST			0.158			0.588
≤ 40 (U/L)	27 (45.8%)	33 (58.9%)		7 (43.8%)	7 (53.8%)	
> 40 (U/L)	32 (54.2%)	23 (41.1%)		9 (56.2%)	6 (46.2%)	
ALT			0.322			0.379
≤ 50 (U/L)	42 (71.2%)	35 (62.5%)		10 (62.5%)	6 (46.2%)	
> 50 (U/L)	17 (28.8%)	21 (37.5%)		6 (37.5%)	7 (53.8%)	
Albumin			0.542			0.321
> 40 (g/L)	10 (16.9%)	12 (21.4%)		5 (31.2%)	2 (15.4%)	
≤ 40 (g/L)	49 (83.1%)	44 (78.6%)		11 (68.8%)	11 (84.6%)	
Total bilirubin			0.403			1
≤ 17.1 (µmol/L)	27 (45.8%)	30 (53.6%)		6 (37.5%)	5 (38.5%)	
> 17.1 (µmol/L)	32 (54.2%)	26 (46.4%)		10 (62.5%)	8 (61.5%)	
Prothrombin time			0.574			0.837
≤ 13 (s)	36 (61%)	37 (66.1%)		8 (50%)	6 (46.2%)	
> 13 (s)	23 (39%)	19 (33.9%)		8 (50%)	7 (53.8%)	
Platelet count			0.412			0.774
≥ 125 × 109/L	25 (42.4%)	28 (50%)		7 (43.8%)	5 (38.5%)	
< 125 × 109/L	34 (57.6%)	28 (50%)		9 (56.2%)	8 (61.5%)	
NLR	3.46 (2.27–5.4)	3 (1.91–5.98)	0.849^b*^	2.38 (1.71–3.35)	4.11 (2.52–6.38)	0.015^b*^
PLR	106 (70–160)	97.5 (67.5–153.3)	0.585^b*^	80.41 (0.467–2.243)	90 (65.24–137.08)	0.589^b*^
*MR imaging features*						
Cirrhosis of background			0.168			1
Absent	17 (28.8%)	23 (41.1%)		6 (37.5%)	5 (38.5%)	
Present	42 (71.2%)	33 (58.9%)		10 (62.5%)	8 (61.5%)	
Tumor number			0.433			0.379
Solitary	37 (62.7%)	39 (69.6%)		6 (37.5%)	7 (53.8%)	
Multiple	22 (37.3%)	17 (30.4%)		10 (62.5%)	6 (46.2%)	
Tumor diameter	4.7 (2.1 to 7. 7)	3.6 (1.7 to 6.12)	0.17^b*^	5.1 (2.02 to 9.65)	4.3 (3.05 to 6.65)	0.559^b*^
Tumor margin			0.321			0.379
Smooth margin	37 (62.7%)	40 (71.4%)		6 (37.5%)	7 (53.8%)	
Non-smooth margin	22 (37.3%)	16 (28.6%)		10 (62.5%)	6 (46.2%)	
Portal venous invasion			0.025			0.044
Negative	43 (72.9%)	50 (89.3%)		9 (56.2%)	12 (92.3%)	
Positive	16 (27.1%)	6 (10.7%)		7 (43.8%)	1 (7.7%)	

Unless indicated otherwise, data are shown as number of patients, with the percentage in parentheses; a, *t*-test; b, Mann–Whitney *U*-test; others (chi-square test or Fisher exact test). *Data are medians, with interquartile ranges in parentheses. NOR, non-objective response; OR, objective response; BCLC, Barcelona Clinic Liver Cancer; AFP, alpha-fetoprotein; CEA, carcinoembryonic antigen; AST, aspartate transaminase; ALT, alanine transaminase; NLR, neutrophils/lymphocytes ratio; PLR, platelet/lymphocytes ratio

### Comparison of forecasting models

Table 2 lists the predictive performance of the four forecasting models where we used area under the ROC curve (AUC), accuracy (ACC), sensitivity and specificity as main measurements. Forecasting models were established using the extracted radiomic features. In the training set, Lasso outperformed others in terms of AUC (0.941) and sensitivity (0.982); DNN had the highest value in terms of prediction ACC (0.870) and specificity (0.864). In the test set, DNN surpassed other models with regard to AUC (0.837), ACC (0.759), and sensitivity (0.923). In the external validation set, the DNN model obtained the best generalization performance with the AUC of 0.796 (accuracy: 0.714, specificity: 0.857). Overall, Lasso and DNN models performed better than KNN and SVM models, which may be due to the ability of DNN and Lasso to select the most important and suitable features automatically. To simplify the research process, KNN and SVM algorithms would not be considered in our subsequent analysis.

**Table 2:** Predictive performance of various models in the training, test and external validation sets

Classifiers	AUC	ACC	Sensitivity	Specificity
Training set				
KNN	0.774	0.704	0.5	0.898
SVM	0.871	0.765	0.891	0.695
Lasso	**0.941**	0.861	**0.982**	0.780
DNN	0.927	**0.870**	0.911	**0.864**
Test set				
KNN	0.669	0.655	0.538	0.75
SVM	0.688	0.621	0.769	0.563
Lasso	0.745	0.655	0.769	**0.813**
DNN	**0.837**	**0.759**	**0.923**	0.688
External validation set				
KNN	0.615	0.536	0.857	0.357
SVM	0.712	0.679	0.786	0.714
Lasso	0.663	0.679	**0.929**	0.500
DNN	**0.796**	**0.714**	0.714	**0.857**

KNN, k-nearest neighbor; SVM, support vector machine; Lasso, the least absolute shrinkage and selection operator; DNN, deep neural networks; AUC, the area under the receiver operating characteristic curve; ACC, accuracy. Bold represents the highest values of AUC, ACC, sensitivity, and specificity in different data sets

**Fig. 2:**
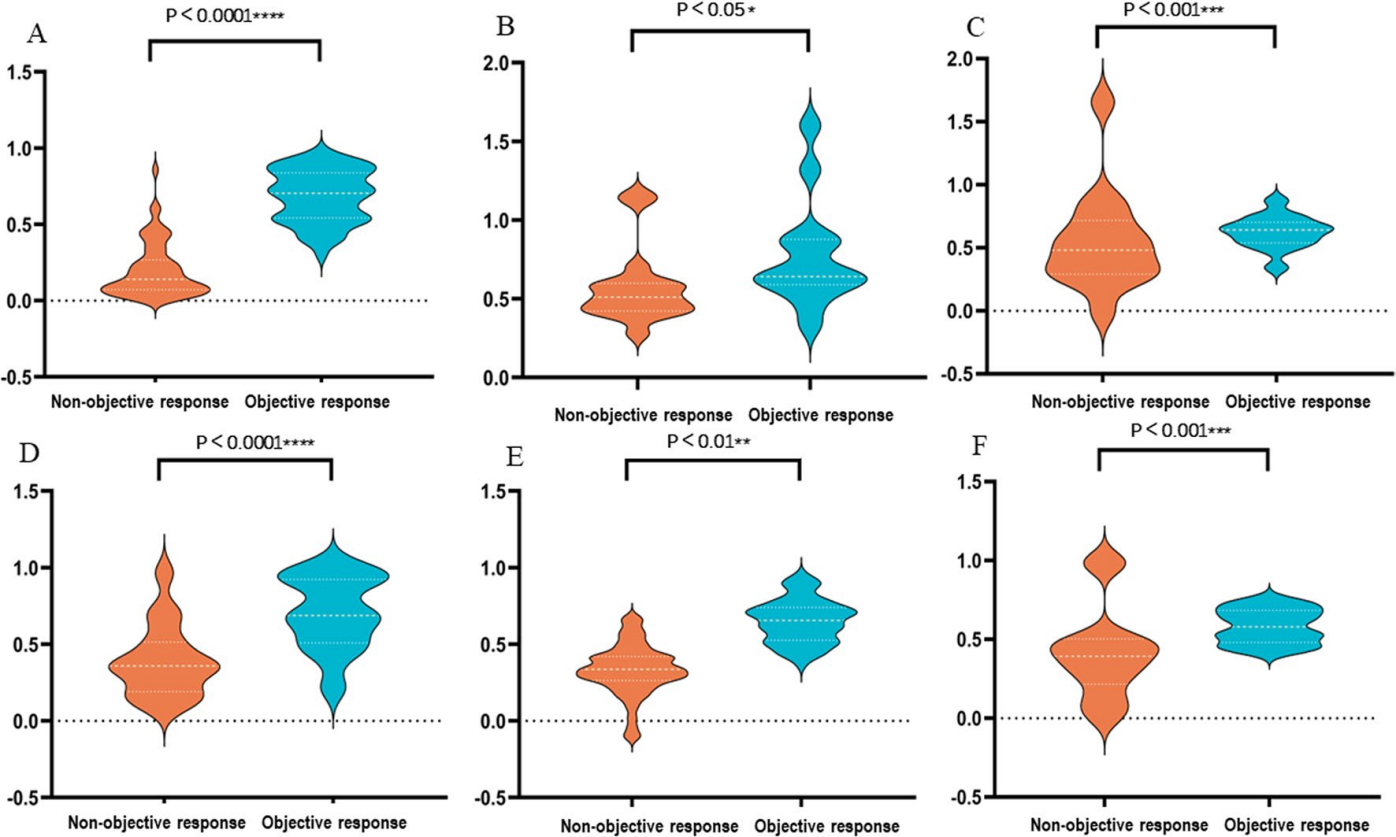
**A** LASSO scores distribution in training set; **B** LASSO scores distribution in test set; **C** LASSO scores distribution in external validation set; **D** DNN scores distribution in training set; **E** DNN scores distribution in test set; **F** DNN scores distribution in external validation set

Then, the trained Lasso and DNN models output the corresponding scores for each patient, and the distribution of scores is shown in Fig. 2. Both Lasso and DNN scores were significantly different between non-objective response and objective response patients in the training set (both *p* < 0.0001), which is further verified in the test set and external validation set. Generally, patients with objective response outcome had higher scores.

### Construction and validation of comprehensive models

**Fig. 3:**
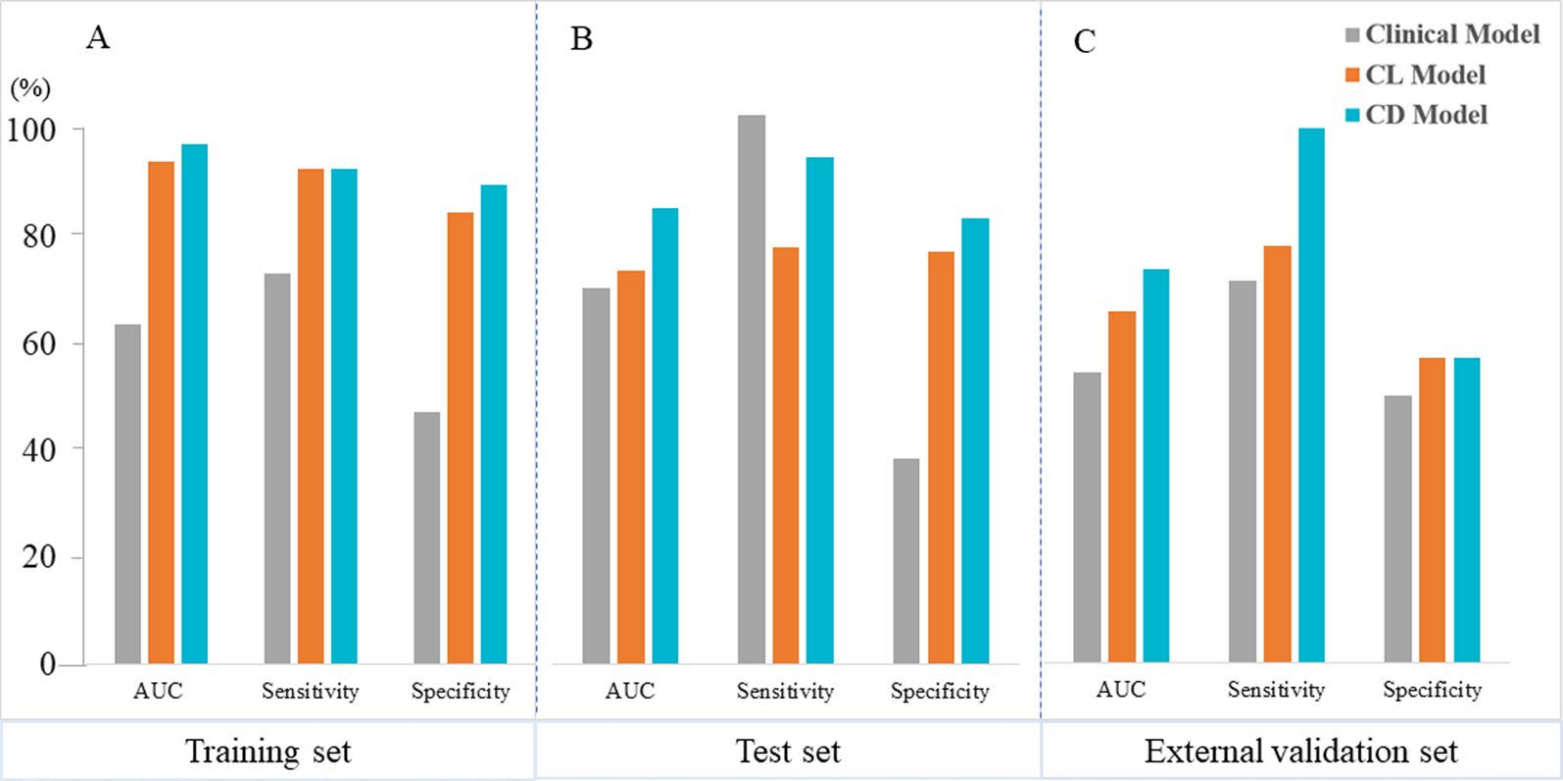
**A** Data for training set; **B** Data for test set; **C** Data for external validation set. Comprehensive models comparison in terms of performance indices for predicting TACE response. Abbreviation: CL model, Clinical & Lasso merged model; CD model, Clinical & DNN merged model; AUC, the area under the curve

To further improve the performance of the model, we developed comprehensive models based on clinical and radiomics features. First, a clinical model was established by incorporating statistically significant variables (i.e., Child–Pugh classification and radiographic venous invasion) and some clinically important variables (such as AFP and AST) as a baseline model for comparison to the comprehensive models. Then, Lasso and DNN scores were combined with clinical indicators to build comprehensive models, namely clinical & Lasso merged model (CL model) and clinical & DNN merged model (CD model). The AUC, sensitivity, and specificity results of the three models are represented in Fig. 3. In the training set, the AUC, sensitivity, and specificity of the clinical model were 0.637, 0.732, and 0.474 respectively; the results of the CL model were 0.943, 0.928, and 0.847, respectively; the CD model performed better, with the AUC, sensitivity, and specificity of 0.974, 0.928, and 0.898, respectively. In the test set, the results of the clinical model were 0.685, 1, and 0.375, respectively; in the CL model, the AUC, sensitivity, and specificity were 0.716, 0.76, and 0.75, respectively; the CD model obtained better AUC (0.831) compared with other models, and the sensitivity and specificity were 0.846 and 0.812, respectively. In the external validation set, CD model generalized well with an AUC of 0.735 (sensitivity: 1, and specificity: 0.571), while the results for clinical and CL model were 0.543 (sensitivity: 0.714, and specificity: 0.5) and 0.658 (sensitivity: 0.78, and specificity: 0.571), respectively.

**Fig. 4:**
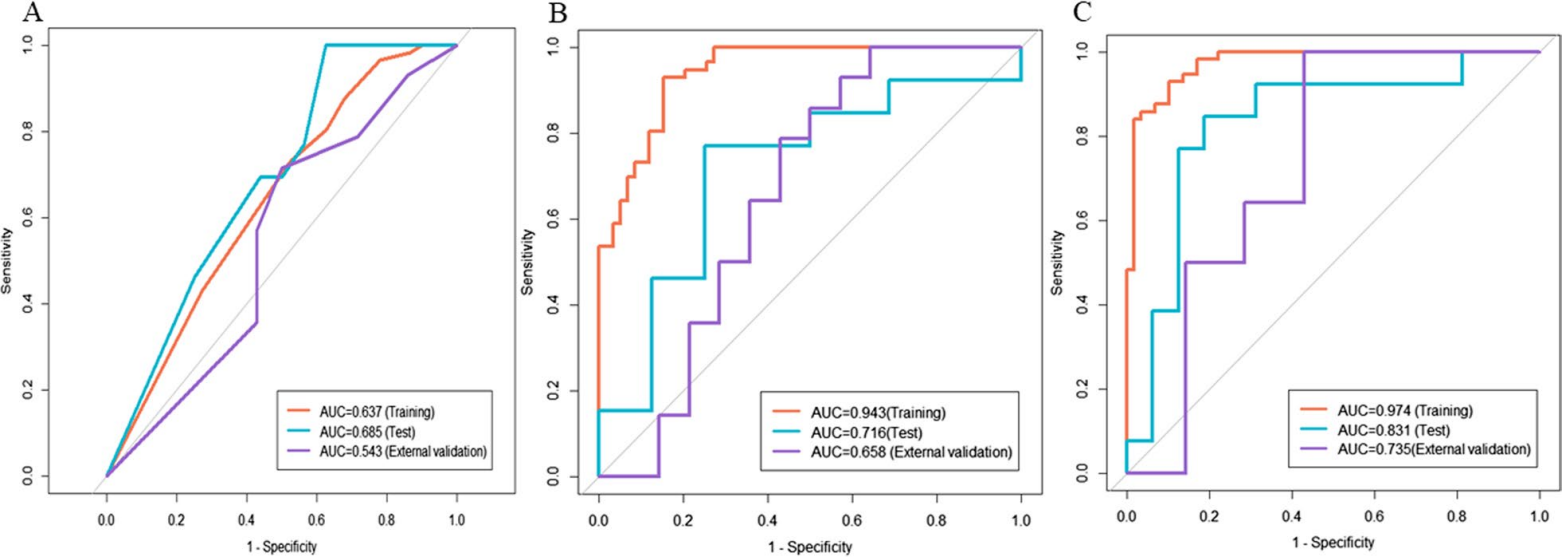
Receiver operating characteristic curve analysis of (**A**) Clinical model, (**B**) CL model (Clinical & Lasso merged model) and (**C**) CD model (Clinical & DNN merged model) for predicting TACE response

**Fig. 5:**
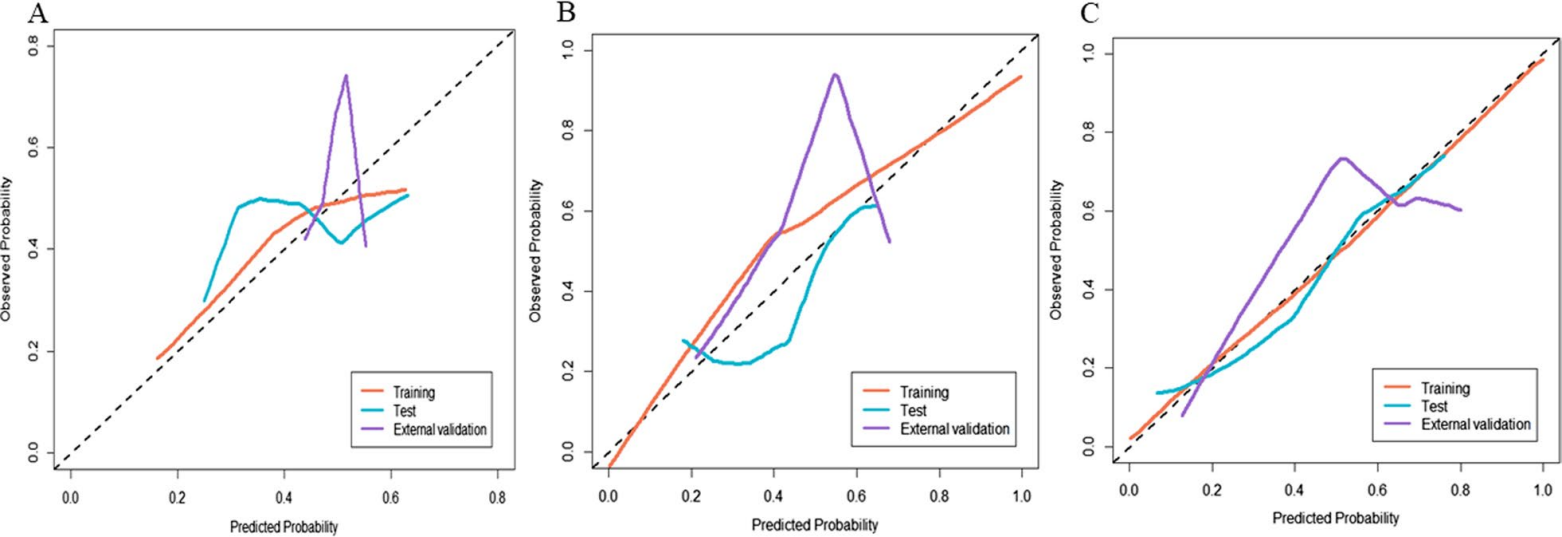
Calibration curves of **A** Clinical model, **B** CL model (Clinical & LASSO merged model) and **C** CD model (Clinical & DNN merged model) for predicting TACE response

**Fig. 6:**
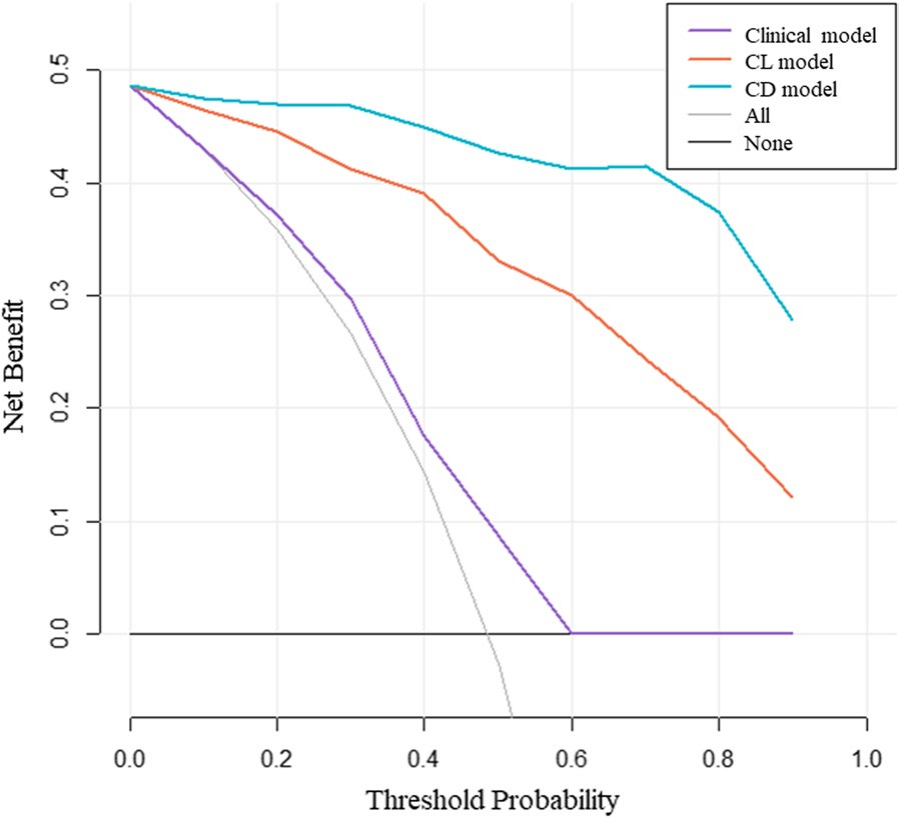
Decision curve analysis (DCA) was used to evaluate the clinical value of the trained models for TACE response. The y-axis measures the net benefit and the x-axis represents the threshold probability. CD model provided the highest net benefit compared with rival models. CL model, Clinical & Lasso merged model; CD model, Clinical & DNN merged model; All, All interventions strategy; None, No intervention at all strategy

The ROCs of the established models are depicted in Fig. 4. In the training set, the CD model was significantly superior to other models (AUC 0.974, 95% CI: 0.951–0.998) according to the results of the Delong test (*p* < 0.05)**.** This performance was further confirmed in the test set (AUC 0.831, 95% CI: 0.667–0.998) and the external validation set (AUC 0.735, 95% CI: 0.529–0.941), indicating that the CD model generalized well in predicting TACE response of unseen new patients. Although the calibration curves portrayed in Fig. 5 show that the consistency between the predicted results and the actual situation needs to be improved, decision curve analysis shown in Fig. 6 demonstrated that CD model provided the highest net benefit compared with rival models.

## Discussion

In this study, we compared the performance of four algorithms in predicting TACE efficacy for HCC patients and found that the Lasso and DNN model performed better. Comprehensive models that integrated with clinically important indicators can significantly improve the prediction performance compared to the baseline clinical model. Among these models, the capability of the CD model (Clinical & DNN merged model) was superior (AUC: 0.974), which was further confirmed by the test set and the external validation set.

Concerning clinical factors, we found that Child–Pugh classification and portal venous invasion were significantly associated with the initial treatment outcome. Child–Pugh classification, which is used for measuring preserved liver function, may help guide treatment selection for HCC patients [33, 34]. Moreover, patients with positive portal vein invasion status tended to gain unfavorable TACE outcomes in our study. This also accords with previous observations [35], which indicated that portal vein invasion was a strong risk factor for TACE. Although in theory, HCC with portal venous invasion is regarded as a contraindication to TACE, many researchers [35–37] have concluded that TACE can be securely and practicably performed in HCC patients with portal vein invasion. Portal vein invasion is not an absolute contraindication of TACE. Therefore, interventional physicians are required to perform individualized assessments based on the portal vein invasion status of different patients to develop personalized treatment plans.

Previous studies have explored the performance of radiomics and deep learning in clinical diagnosis, therapy strategy, and prognostic assessment in the realm of oncology [38–41]. Indeed, Kong et al. have previously conducted an investigation using MR images to predict TACE response, but the outcome was not satisfactory enough with the highest AUC of 0.884. In terms of image input, previous studies only adopted single-sequence images (i.e., T2WI) to train the model [12]. With four sequences of MR images as inputs, this research improved the AUC from 0.812 to 0.941 compared with the previous study. This suggests that different sequences of MR images may provide more information and further improve the predictive performance of the model. Besides, there was only a single mathematical model involved in the procedure and the final proposed model was not good enough (AUC = 0.861). Therefore, it is imperative to specifically compare different potential algorithms and pick out the most robust one.

In present study, we compared the performance of four classifiers in predicting TACE response for HCC patients. The prediction performance of the DNN and Lasso models was superior to other forecasting models when using the same extracted feature inputs. The performance of DNN and Lasso classifiers was similar in the training set. However, both the AUC and ACC of the DNN in the test set and external validation set were significantly higher, indicating that the generalization ability of DNN model may surpass that of Lasso model. Although the LASSO and DNN models can achieve relatively satisfactory performance, the role of important non-radiomics variables in the prediction model cannot be ignored [42]. Therefore, we established comprehensive models that integrating clinical information and feature classifiers. Both CL and CD models displayed improved predictive performance compared with the baseline clinical model, while the optimization efficacy of CD model was better. Specifically, the CD model increased the AUC value from 0.637 to 0.974 in the training set. This was also confirmed in the test set (AUC value increased from 0.685 to 0.831) and the external validation set (from 0.543 to 0.735), showing that the CD model obtained good robustness in predicting TACE response of unknown new cases. Similarly, previous investigations [43, 44] demonstrated that DNN performed better than other conventional (such as SVM or Lasso) methods in predicting clinical endpoints. A possible explanation for this might be that DNN can prevent network over-fitting with the help of BatchNorm and Dropout modules [30, 31]. On the other hand, DNN can realize automatic assignment of proper weights to each parameter based on its contribution with no dimensionality reduction required, thereby incorporating different large data very effectively [45].

Our study had several limitations. First, the subjects included were relatively limited, which may lead to selection bias. The calibration ability of the proposed model was not satisfactory enough, which may be related to the selection bias. Hence, more samples are needed to be involved to optimize the model. Still, according to the decision curve analysis of the model, the net benefit of the CD model is significantly higher than that of the simple clinical model and the CL model, indicating that the engagement of the CD model to assist decision-making is more clinically practical. Secondly, this research was based on the data of a single institution, and multicenter investigations are required to further demonstrate the generalizability of the proposed model and optimize precise medical management for TACE treatment.

In conclusion, the results of the model-performance comparisons in this study indicate that the DNN model is the most clinically useful method in predicting TACE response for HCC. After integrating with clinically significant factors, the proposed CD model (Clinical & DNN merged model) may be valuable in pre-treatment counseling of HCC patients who may benefit the most from TACE.

## Data Availability

The datasets generated and/or analyzed during the current study are not publicly available but are available from the corresponding author on reasonable request.

## Abbreviations

ACC: Accuracy
AI: Artificial intelligence
AUC: Area under the curve
CE-MR: Contrast-enhanced magnetic resonance
DNN: Deep neural network
HCC: Hepatocellular carcinoma
ICC: Intraclass correlation coefficient
KNN: K-nearest neighbor
Lasso: The least absolute shrinkage and selection operator
mRECIST: The modified Response Evaluation Criteria in Solid Tumors
MRI: Magnetic resonance imaging
ROC: Receiver operating characteristic
SVM: Support vector machine
VOI: Volume of interest
